# Hyaluronan and RHAMM in Wound Repair and the “Cancerization” of Stromal Tissues

**DOI:** 10.1155/2014/103923

**Published:** 2014-08-04

**Authors:** Cornelia Tolg, James B. McCarthy, Arjang Yazdani, Eva A. Turley

**Affiliations:** ^1^London Regional Cancer Program, London Health Sciences Centre, Room A4-931A 790 Commissioners Road East, London, ON, Canada N6A 4L6; ^2^Department of Laboratory Medicine and Pathology, Masonic Comprehensive Cancer Center, Minneapolis, MN 55455, USA; ^3^London, ON, Canada N6A 4V2 Division of Plastic and Reconstructive Surgery, University of Western Ontario, London, ON, Canada N6A 4V2

## Abstract

Tumors and wounds share many similarities including loss of tissue architecture, cell polarity and cell differentiation, aberrant extracellular matrix (ECM) remodeling (Ballard et al., 2006) increased inflammation, angiogenesis, and elevated cell migration and proliferation. Whereas these changes are transient in repairing wounds, tumors do not regain tissue architecture but rather their continued progression is fueled in part by loss of normal tissue structure. As a result tumors are often described as wounds that do not heal. The ECM component hyaluronan (HA) and its receptor RHAMM have both been implicated in wound repair and tumor progression. This review highlights the similarities and differences in their roles during these processes and proposes that RHAMM-regulated wound repair functions may contribute to “cancerization” of the tumor microenvironment.

## 1. Introduction

Tumors have often been compared to chronic wounds that do not heal. The tumor microenvironment, which is a critical but incompletely understood factor in promoting tumor progression, exhibits tissue remodeling characteristics similar to wounds. These include loss of cell polarity/tissue architecture and remodeling (degradation/resynthesis and reorganization) of the ECM [1], as well as cell dedifferentiation, migration, and proliferation [2–7]. A prolonged and episodic remodeling of adult tissue that results in loss of architecture is also associated with an increased susceptibility for tumor initiation. For example, gestation and involution in breast tissue, which are two periods of prolonged and repeated mammary tissue remodeling, are both linked to increased breast cancer (BCa) susceptibility [8–15].

**Figure 1:**
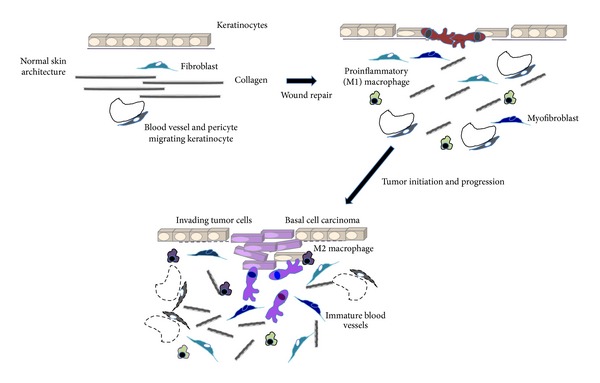
Schematic summarizing wound and tumor microenvironment remodeling in skin. The normal tissue architecture of skin is well-organized in both the epidermis, which consists of differentiated cohesive keratinocytes, and the dermis, which is composed of fibroblasts, blood vessels, and well-organized collagen fibrils amongst other ECM components. Tissue injury results in temporary changes in tissue architecture as keratinocytes dedifferentiate and migrate across wound gaps, proinflammatory macrophages migrate into the dermis, angiogenesis is promoted, and subpopulations of fibroblasts differentiate into myofibroblasts that organize collagen fibrils, which contribute to scar tissue. Tumor initiation also results in dedifferentiation, proliferation and migration/invasion of keratinocytes, influx of macrophages, differentiation of fibroblasts into myofibroblasts that increase deposition and scar like organization of collagen fibrils, and formation of new immature blood vessels. However, this disorganized tissue architecture is not transient as it is in wound repair but increases with tumor progression.

Most adult wounds heal by fibrosis, which is characterized by an inflammatory response, changes in the composition of ECM, accumulation of biologically active ECM fragments, and scarring [16–20]. There are also accompanying changes in the cellular content of the wound environment that include the differentiation of myofibroblasts, which contribute to wound closure, the formation of a microvasculature, collagen I deposition, and scarring [17, 21–26]. Finally, there is an infiltration of circulating fibroblasts [27–29] and innate immune cells [30] that synthesize and ultimately contribute to repair completion and restoration of tissue architecture. Aspects of this fibrotic milieu provide a protumorigenic microenvironment that enhances both tumor initiation and expansion [31–34]. For example, the presence of high density or fibrotic regions in breast, often resulting from radiation treatment, are sites commonly associated with tumor recurrence [35, 36]. This observation and others suggest a model for tumor initiation that is associated with the chronic or frequent (e.g., episodic) loss of normal tissue architecture and wound-like ECM remodeling, which enhances rogue behavior of mutant cells by providing a “cancerized” microenvironment (Figure 1) [37, 38]. Once tumors are initiated, molecular mechanisms associated with malignant progression function in a dynamic and reciprocal manner with host cells to sustain and enhance this protumorigenic wound-like microenvironment. It should therefore be no surprise that gene signatures and transcriptomes of tumors are enriched in wound repair profiles and that these profiles are associated with or prognostic of poor outcome [39–44].

Quite often discussion on the importance of ECM remodeling in wound repair and protumorigenic stroma focuses upon alterations in the synthesis and fragmentation of ECM proteins [45–47]. However, a consideration of the tissue polysaccharide HA is usually not included in these discussions, despite the fact that elevated HA production is essential for tissue repair, is required for tumor progression in numerous experimental models, and is linked to poor outcome in many cancers including BCa [3, 30, 48]. Therefore the first part of this review will focus on HA metabolism as it relates to wound healing and BCa initiation/malignant progression. There is also clear and convincing evidence that HA receptors such as cluster designation 44 (CD44), receptor for hyaluronan mediated motility [49], and toll-like receptors 2,4 (TLR2,4) (to name a few) are all important contributors to malignant progression and outcome in BCa patients. There are many excellent reviews on the functions associated with these and other HA receptors in tissue homeostasis, wound repair, and tumor 4 progression [3, 30, 48, 50–53]. However, this review will focus on the multifunctional HA receptor, RHAMM (gene name HMMR), because of its clear roles in fibrotic wound repair that are apparently relevant to BCa initiation and progression. For example, the expression levels of HMMR/RHAMM are frequently increased in BCa and linked to poor clinical outcome [54] and considerable* in vivo* evidence links RHAMM expression levels to mesenchymal response-to-injury [55–60]. The disparate functions of RHAMM are related to its complex subcellular localization. RHAMM was originally described in the context of one of these functions, which is to facilitate HA mediated cell motility [48, 61–64] but was more recently shown to affect centrosomal function and mitotic spindle integrity. The purpose of this review is to highlight the roles of HA and RHAMM that converge in wound repair and BCa progression.

## 2. Hyaluronan Background

HA is a polysaccharide belonging to the glycosaminoglycan family of macromolecules. This biopolymer consists of repeating disaccharides of N-acetylglucosamine and *β*-glucuronic acid, the latter of which confers anionic properties to HA [51, 53, 65]. There are three known hyaluronan synthases (HAS1-3) that produce HA and these are differentially expressed during wound repair and in tumors [48, 66–78]. HAS enzymes are proteins that contain multiple membrane spanning sequences and to date they are the only known glycosyltransferases imbedded in the plasma membrane. “Activated” UDP-sugars are sequentially added to the catalytic portion of the enzyme located on the inner face of the plasma membrane. The synthesized polymer is then extruded through the plasma membrane, possibly through channels created by oligomerization of the synthases [76, 79].

HA is a ubiquitous component of tissue ECM but is found in particularly high concentrations as a native homeostatic form within hydrated tissues such as the vitreous of the eye, articular cartilage, and lymphatics and skin. It is particularly enriched in the epidermis, where it is important for maintaining the hydration of this tissue so that it can form a more effective barrier to the environment [80–82]. During embryonic development HA is a crucial component of cardiac jelly and is absolutely required for heart development where it provides a migration-supporting environment for cardiac cushion cells [83]. Genetic deletion of HAS2, the HA synthase that is responsible for HA synthesis during the embryonic developmental period when heart development occurs, results in embryonic lethality as a result of defective cardiac development [84]. Genetic deletions of HAS1 or 3 do not have the same developmental consequences emphasizing that these synthases are expressed differentially. They also exhibit distinct spatial distributions in tissues [68, 85–87]. The biological significance of having 3 unique but closely related HA isoforms is not completely understood, but in addition to differential mechanisms regulating their expression, they also synthesize HA biopolymers of dissimilar average sizes. Such differences in polymer size are linked to distinct HA functions [48, 65, 88–90]. Thus, it is not surprising that there are temporal, spatial, and cell-type specific differences in HAS1-3 expression during tissue repair and in tumor progression. For example, during repair of excisional wounds, keratinocyte migration is associated with elevated HAS2 and 3 expression while peritoneal mesothelial cells upregulate only HAS2 following mechanical injury [69, 72]. There is surprisingly little information about HAS expression and HA production during tissue remodeling that is not associated with injury. For example, although branching morphogenesis in general is known to be HA dependent, to our knowledge its role in mammary gland morphogenesis has not been reported. A great deal more is known about the roles of HA and HAS isoforms in the initiation and progression of tumors from breast tissue. HAS1 and HAS2 expression are commonly upregulated in BCa. Their elevated expression and HA accumulation are linked to ominous features of malignant progression. This includes epithelial-to-mesenchymal transformation (EMT) [91–93] and increased invasion [3, 54, 94–96], providing a partial explanation for HA's relationship to poor outcome [48, 74, 93, 97].

**Figure 2:**
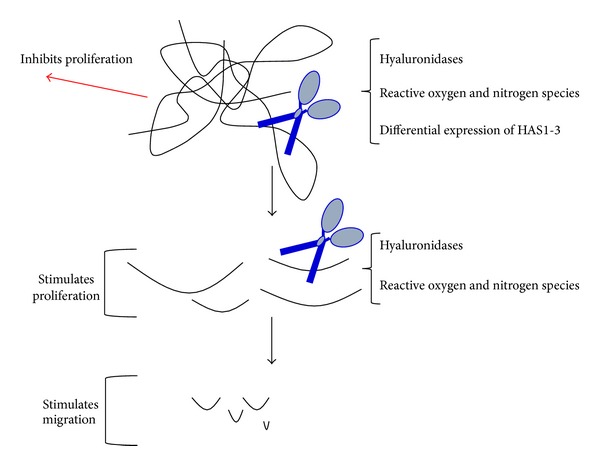
HA functions are molecular weight dependent. HA occurs as a large native polymer in homeostatic tissues but is degraded following tissue injury by free radicals and hyaluronidases. The resulting fragments have different bioactivity than the native polymer depending upon their size. For example, intermediate fragments can stimulate cell proliferation while smaller fragments have been reported to only promote cell migration.

Glycosaminoglycans such as HA bind, concentrate, present, and prevent diffusion of growth factors in tissues. HA is fundamentally important for both maintaining tissue homeostasis and orchestrating the inflammatory, fibrotic, and stem cell renewal responses in damaged tissues. High molecular weight HA is typically produced by homeostatic tissues and can reach up to 2,000 kDa. High molecular weight HA performs ECM and growth factor presenting/scaffolding functions and is critical for tissue hydration [81, 98]. By providing a relatively loose matrix, HA supports cell migration, acts as scavenger for reactive oxygen and nitrogen species, and is typically anti-inflammatory [30, 50, 65, 99]. Importantly, recent findings also suggest that high molecular weight HA exhibits tumor suppressive functions in skin [100]. By sharp contrast, the fragmentation of polymeric HA, which occurs during both normal and disease-associated tissue remodeling, drastically alters the functions of HA. HA fragments are typically proinflammatory and promigratory and promote proliferation (Figure 2). This change in function as a result of depolymerization is similar in principle to the fragmentation of certain embryonic morphogens and extracellular matrikines which have distinct biological properties compared to the intact molecules, and which occur during tissue remodeling [98]. HA fragmentation can occur as a result of hyaluronidase activity (e.g., Hyal1 and Hyal2) or the presence of reactive oxygen and nitrogen species [81, 98]. Furthermore, HA size heterogeneity within a wound can also result from altered expression levels of distinct HAS isoforms [48]. Localized changes in HA synthesis and fragmentation may therefore represent a type of “on-off switch,” which is important for providing early warning signals of tissue damage, requiring host cells to respond appropriately to restore tissue function and architecture [50, 98, 101–105]. It needs to be emphasized, however, that the majority of these studies have been performed in culture with very few analyses determining the extent of HA fragmentation in intact tissues during tissue remodeling* in vivo*. Therefore aspects of these* in vitro* studies that are applicable to tissue remodeling* in vivo* have yet to be fully defined.

The development of new techniques [106, 107] that facilitate the isolation and determination of HA fragments size distributions from complex tissues will ultimately provide an important framework for understanding the biological importance of size heterogeneity of this biopolymer. While* in vivo* analysis of these fragmentation patterns is just the beginning, there is mounting evidence* in vitro* that the biological impact of HA size heterogeneity is related to the ability of different HA receptors to bind high molecular weight polymeric versus HA fragments. As an example, while higher molecular weight HA binds to CD44, smaller fragments of HA bind to RHAMM and even smaller fragments bind to TLR 2,4. There is evidence that these HA fragments can also function to inhibit the binding of higher molecular weight HA to CD44 [108, 109]. Thus, fragmented HA could impact tissues by either directly binding specific receptors or antagonizing the binding of larger HA polymers to their cognate receptors. These complex interactions control a variety of signaling pathways that regulate cell adhesion/motility, mitotic spindle formation, and transcriptomes. Clearly much work is needed to dissect the complex roles of native HA versus fragments in normal and diseased tissues, with longer term impact of providing specific targets associated with pathologies linked to altered HA metabolism.

## 3. Hyaluronan and Tissue Remodelling

The importance of the synthesis/fragmentation of high molecular weight HA has been most extensively studied in models of tissue repair. HA is considered a “keystone” or central molecule in regulating response to tissue stress since rapid alterations in HA production, macromolecular organization, and size within tissues are among the earliest changes that can be detected following injury including those resulting from exposure to ionizing radiation [30, 81, 98, 110, 111]. Like other ECM components (e.g., collagen), HA is fragmented during wound repair into small proinflammatory oligosaccharides by oxygen/nitrogen free radicals that accumulate in the stressed tissue [112, 113]. These wound fragments constitute an early “danger signal” that is part of the damage-associated molecular process (DAMP). DAMP stimulates the innate immune response, which, when not resolved, directly contributes to chronic inflammation and tissue fibrosis [106, 114–117]. Experimental models of tissue injury have documented the contribution of HA to DAMP and to the repair of excisional skin wounds [81, 118], vascular response to injury [119, 120], and induced lung injury [30]. The paradigm for the functions of HA in all of these injured tissues is similar, and recent reviews have summarized the literature in both vascular and lung injury models [110, 121]. Our studies on the identification and characterization of HA size heterogeneity in excisional wound healing are among the first to attempt to shed light on these issues in skin wounds* in vivo* [50, 106]. These studies have demonstrated an association between HA fragmentation and cellular infiltration into wounded tissues [56, 122, 123]. Furthermore they have shown that this cellular infiltration is defective in animals that are embryonic null for RHAMM [55, 56].

HA occurs in large amounts in skin and it is a key factor in its homeostasis since it controls both fibroblast differentiation and epidermal activation and renewal [118, 124]. As in other tissues, native, high molecular weight HA suppresses skin inflammation [30, 118, 125], myofibroblast differentiation [125], and fibrosis [118]. Similar to other injured tissues, fragmentation of HA, which results from ionizing radiation and other damage, promotes inflammation and tissue fibrosis. Homeostatic skin contains the largest depot of high molecular weight, anti-inflammatory (native) HA in the body and this is primarily organized into extracellular macromolecular complexes in the dermal ECM. It is also detected as pericellular coats, which are particularly noticeable around keratinocytes [126–128]. These HA coats, which are sustained on keratinocytes surfaces via the HA receptor CD44 [129], are required for maintenance of barrier/permeability functions of skin as well as for keratinocyte renewal, proliferation, and differentiation [81, 130–133]. Because of their exposure to the environment, a key additional homeostatic function of keratinocyte-associated coats is that they protect against ionizing radiation-induced DNA damage. For example, production of native HA protects against DNA damage resulting from either UVB or gamma radiation [81]. Native HA in skin is also linked to reduced risk of cancer (skin) and metastasis [92, 100]. Importantly, loss of keratinocyte HA coats is linked to epidermal atrophy and other pathologic lesions including increased dermal fibrosis [124]. In addition to its effects on keratinocytes, skin HA metabolism also controls proinflammatory immune cell influx [30] and TGF*β*-1 induced myofibroblast differentiation, which is largely responsible for fibrosis [118, 125]. Reepithelialization of injured skin begins early after injury and is controlled by EGF signaling, which stimulates HA production and regulates promigratory/proliferation signaling through HA and CD44 [134]. TGF*β*-1 is strongly antiproliferative in these cells [135].

Reepithelialization serves as a critical function in maintaining the integrity of the dermal layers due to crosstalk between these two skin layers. Although not a great deal is known about this function, TGF*β*-1/SMAD 3 signaling plays a key role. Thus, when wound repair is deregulated and reepithelialization is prevented, dermal fibrosis is enhanced [136]. The epithelium in transgenic mice, which are engineered to suppress SMAD 3, exhibits accelerated reepithelialization, reduced inflammation, and reduced dermal fibrosis [137]. Reepithelialization of wounds normally coincides with and may instruct removal of dermal myofibroblasts by apoptosis. Blocking reepithelialization prevents myofibroblast apoptosis and results in hypertrophic scars or chronic tissue fibrosis [124]. HA, which as noted above is necessary for keratinocyte proliferation and migration in response to epidermal injury, is also a key regulator of TGF*β*-1 functions in fibrosis and myofibroblast differentiation [118, 125].

Fibroblast differentiation into myofibroblasts is controlled by two cooperating pathways, TGF*β*-R/SMAD and HA mediated RHAMM:CD44:EGFR signaling complexes, both of which must be activated to induce myofibroblast differentiation [138]. Thus, the TGF*β*-1 signaling pathway promotes HAS2 dependent HA synthesis and pericellular HA coat formation. This results in increased levels of endogenous TGF*β*-1, which maintain myofibroblast differentiation via an autocrine loop consisting of HA:TGF*β*-1 production [139]. TGF*β*-1 and HA dependent signaling promote CD44 and EGFR interaction in lipid rafts resulting in the activation of ERK1,2 and calmodulin kinase II activation [138]. Intriguingly, increased accumulation of native extracellular HA [118] such as what occurs during repair of embryonic tissues [140, 141] or disruption of HA:RHAMM:CD44 [106] complexes in wounds can negatively regulate these signaling pathways so that myofibroblast differentiation is reduced and, conversely, reepithelialization is promoted. The role of HA in wound myofibroblast differentiation is important since these cells closely resemble cancer associated fibroblasts that are intimately involved in development of a cancerized microenvironment that facilitate the progression of BCa (e.g., [142]).

Studies implicating differential roles for high molecular weight versus fragmented HA in the repair of other tissues prompted us to further investigate the potential relationship between HA synthesis and size heterogeneity in excisional wound repair [30, 98]. As anticipated, homeostatic adult mouse skin contains mainly high molecular weight HA (>5,000 kDa) while fragmentation can be detected in wounds as rapidly as 24 h, peaking at 7 days after injury [106]. Importantly high molecular weight polymers (>5,000 kDa) as well as intermediate-to-small (30–500 kDa) and very small (<10 kDa) HA fragments coexist in these wounds. This fragmentation pattern is consistent with an important role for HA size heterogeneity in promoting excisional dermal wound repair. HA fragments signal as “on” switches to both circulating and resident cells, which promote inflammation and angiogenesis while coexisting high molecular weight polymers signal as “off” switches that limit responses to fragments. In support of this notion, it has been shown that either forced HAS1 expression or supplementing the wounds with high molecular weight HA limits the fibrotic response such that the healing of these wounds more closely resembles those observed in embryos. The effect of adding excess high molecular weight HA to these wounds leads to reduced levels of TGF*β*-1, attenuated inflammation, and a reduction in biomechanical stress [22, 143, 144]. A number of studies have established that small to intermediate size HA fragments promote the influx of immune cells. Such fragments also activate the proinflammatory functions of these infiltrating cells, which include stimulating expression of chemokines such as MIF-1a and MCP and increasing the expression of profibrotic growth factors such as TGF*β*-1 [30]. This size range of HA fragments also promotes branching morphogenesis of wound associated blood vessels [119] and activation of fibroblasts [56, 145].

The availability of precisely or at least restricted sizes of small HA polymers produced by recombinant technology has allowed studies that suggest a much more complex functional repertoire and interplay of high molecular weight HA versus HA fragments than previously suspected. For example, a range of HA fragment sizes (e.g., HA-12 (12 saccharides) and HA-880 (880 saccharides) and native, high molecular weight HA) all activate ERK1,2, Akt, and P38 signaling cascades and all increase expression of ECM remodeling proteinases such as MMP1,3 [122, 146–150]. However, HA-12 and native HA selectively promote expression of collagen III and TGF*β*-3 and HA-12 solely promotes TIMP1 expression by dermal fibroblasts in culture [56, 149]. HA-6 (6 saccharides) but not HA-8, HA-10, 40 kDa, or native HA stimulates wound closure and increases wound macrophages and TGF*β*-1 levels. In spite of stimulating TGF*β*-1, HA-6 does not increase myofibroblast differentiation suggesting requirement of additional stimuli and possibly other HA fragment sizes [56]. These studies serve to emphasize the enormous amount of information that is generated by differential fragmentation of HA associated with tissue injury. The mechanism by which this information is transduced to the cell is currently not well understood. For example, are the trafficking/display patterns [54] of HA receptors that have differential binding properties for discrete sizes of HA fragments involved in signal transduction of the HA fragment pool as a whole? Furthermore, is there a temporal or spatial relationship between HA fragmentation and the response of specific cellular subpopulations expressing various HA receptors? Despite the complexity, an important first step is to document the presence and kinetics of HA fragmentation patterns in order to address these and other questions in the future. These analyses of HA functions in excisional wounds and other models of tissue injury have uncovered an information-rich mechanism for finely regulating the key processes of inflammation, angiogenesis, and fibroblast activation that are all essential for efficient wound repair [98]. These same processes in the tumor microenvironment appear to be required for the initiation and progression of BCa.

## 4. Stromal Hyaluronan and Breast Cancer 

In BCa it is clear that tumors progress more aggressively in a HA-rich microenvironment and that stromal HA affects both host and tumor cells to accelerate progression. Many of the functions of HA during BCa progression have been summarized in several recent excellent reviews [3, 48, 78, 97, 125]. The importance of stromal HA in mediating host responses, which support BCa progression, is the focus in this section.

Both tumor parenchyma and host cells in the tumor microenvironment express HAS isoforms and produce HA, which then accumulates in tumor parenchyma and in the peritumor stromal tissues [48, 97]. Clinical studies suggest that HA accumulating in either the tumor parenchyma or surrounding peritumor stroma is tightly linked to BCa progression and both are independent prognostic indicators of poor outcome [97]. Stromal cells, in particular cancer associated fibroblasts, express all HAS isoforms. Increased HAS expression by cancer associated fibroblasts correlates with increased HA accumulation, increased stromal CD44 expression, high relapse rate, and short overall survival [74]. Furthermore, high stromal HA accumulation is significantly associated with the appearance of a tumor reactive stroma, which associates with tumor cell positive lymph nodes, high tumor grade, and lymphatic tumor emboli [151]. The increased expression of extracellular HA binding proteins such as versican has also been reported [152]. Analyses of head and neck tumors reveal previously unappreciated stromal cancer associated fibroblast heterogeneity involving HA: a subpopulation of cancer associated fibroblasts produces high levels of HA and promotes local tissue invasion by cancer cells [153].

Bigenic expression of Neu and HAS2 in ductal epithelium using the MMTV promoter in mice results in marked changes in the peritumor stroma resembling those observed clinically in tumor reactive stroma [154]. Furthermore, these studies show that HA produced by the tumor parenchyma by itself can enhance stromal cell recruitment and the formation of a tumor reactive stroma. Notable phenotypic changes include increased formation of intratumoral HA-rich stroma, accumulation of ECM components, such as versican, collagen 1, and fibronectin, neovascularization, and the infiltration of immature mesenchymal cells. Cytokine analyses suggest that the increased accumulation of these stromal cells stimulates neoangiogenesis. Coinjection experiments with cancer associated fibroblasts that are derived from these bigenic tumors reveal that the cancer associated fibroblasts are responsible for the observed increase in tumor growth, reactive stroma formation, angiogenesis, and lymphangiogenesis [155]. Others have shown that tumor-associated HA supports tumor cell epithelial-mesenchymal transformation that also enables the growth and spread of tumor cells [154–157]. Collectively these studies indicate that production of HA by tumor cells favors recruitment of mesenchymal cells that remodel the peritumor stroma to create a tumor friendly microenvironment.

In addition, microenvironments rich in HA provide mitogenic and motogenic signals for tumor cells. For example, we have shown that human BCa lines are heterogeneous in their ability to bind to HA and that exposure to this glycosaminoglycan promotes specific subpopulations within these lines to divide rapidly while stimulating other subpopulations to invade aggressively but proliferate slowly [54]. This type of functional heterogeneity may be partly responsible for the relationship between HA-rich tumor microenvironments and relapse/poor outcome reported by a number of clinical studies. Elevated stromal HA is linked to HER2 positivity and several key clinicopathologic features including poor prognosis factors such as tumor size, lymph node positivity, hormone receptor negativity, increased relapse rate, and shortened survival [158]. The mechanisms by which stromal HA effects BCa progression and the roles of native versus fragmented HA are not yet well understood. However, considering the evidence from multiple model systems (discussed above) it is clear that these effects are mediated through HA receptors. Multiple HA receptors (e.g., CD44, RHAMM and LYVE-1, and TLR2,3 among others) are known to be involved in BCa progression [3, 48, 156, 159–161]. The role of RHAMM in progression of this disease and in wound repair will be considered here because RHAMM is unique in the ways in which it converts HA “signaling” into multiple key aspects of cellular functions that are relevant to response to injury and to tumor progression.

## 5. RHAMM Background

**Figure 3:**
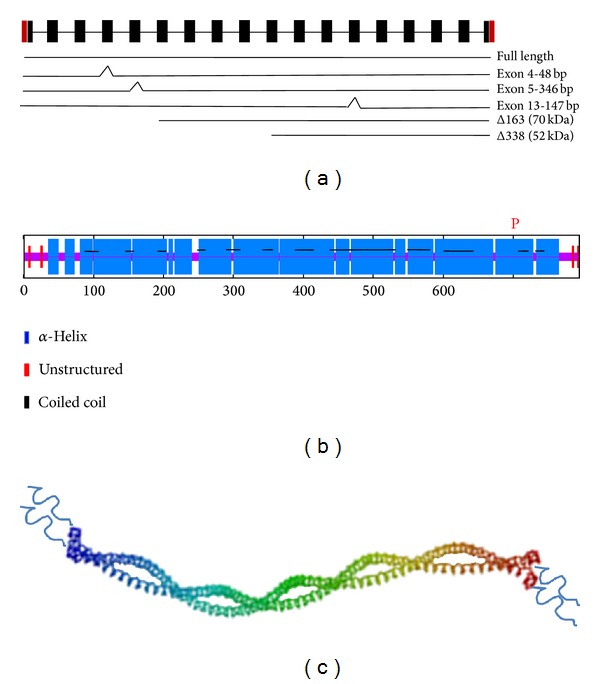
RHAMM isoforms, protein secondary structure, and posttranslational modification (a) RHAMM exon structure is shown as black boxes. Lines underneath this diagram show the known isoform structures. The full-length protein (85 kDa in human) is largely associated with interphase microtubules and the mitotic spindle during the cell cycle. Three isoforms are generated by alternative splicing of exon 4, 5, or 13. Loss of exon 4 sequence disrupts stable association with interphase microtubules and results in the appearance of RHAMM in the interphase cell nucleus. N-terminal truncations that may be generated by posttranslational mechanism or alternative start codon usage are very transiently expressed during early tissue injury but are constitutively present in some aggressive breast cancer cell lines and tumors. These accumulate in the nucleus and on the cell surface. (b) RHAMM protein is predicted to be largely a-helical, with unstructured sequences at the extreme N and C-termini. The orange P at the carboxyl terminus indicates an AURKA and ERK1,2 phosphoacceptor site. RHAMM also contains approximately 30 putative protein kinase C phosphoacceptor sites (not shown). This posttranslational modification is associated with the nuclear accumulation of RHAMM. Although RHAMM is phosphorylated by protein kinase C, the acceptor sites have not yet been reported. Protein kinase C modification of RHAMM is linked to interphase centrosomal placement. (c) The secondary structure predictions shown in (b) indicate that RHAMM proteins can self-associate to form random coiled coils.

Studies using RHAMM null animals have clearly established an important role for this protein in tissue response to injury [55, 57]. Furthermore, a number of studies have linked RHAMM expression to BCa since it is frequently elevated in breast and other cancers and is associated with poor outcome [54, 61, 162]. RHAMM is a largely hydrophilic helical protein (Figure 3) that was originally isolated from conditioned medium from chicken heart explant cultures exhibiting high HA production and increased cell migration [163]. It binds to HA via positively charged amino acid clusters in the carboxyl terminus that are structurally distinct from the link module responsible for HA binding to CD44 [164]. RHAMM also binds to microtubules via sequences located in its N- and carboxyl termini. It directly binds to ERK1 via a sequence with homology to a D-box MAP kinase interaction site [95, 165, 166]. The protein contains several leucine zippers and these together with its potential for forming a coiled coil predict that it can self-associate as dimers or trimers (Figure 3).

*In vivo*, RHAMM expression is tightly regulated: it is poorly expressed in most homeostatic adult tissues with the exception of ovaries, testes, and ciliated epithelium of the respiratory tract in which elevated RHAMM mRNA levels are detected [167–169]. However, RHAMM mRNA and protein expression are strongly but transiently increased in response to injury. A number of mechanisms have been identified that either promote or suppress RHAMM expression. Promoting factors include TGF*β*-1, RON, and the YAP-HIPPO pathway while tumor suppressors such as p53 and BRCA1 reduce its expression [49, 61, 96, 162, 170–176]. Analyses of RHAMM knockout mice [57, 58, 177] show that it is perhaps surprisingly not required for embryogenesis or homeostatic adult functions, the latter predicted by its low or absent expression in most tissues [177]. However it is essential for a variety of tissue repair processes that like embryogenesis require cell migration, invasion, and ECM remodeling. Since elevated levels of RHAMM are associated with poor prognosis in human cancers, it would appear that tumor cells usurp these wound repair functions of RHAMM to facilitate their survival and progression. The restricted expression of RHAMM makes it a potential target for cancer and wound repair therapy with low toxicity. Indeed, RHAMM peptides that are currently being tested in phase II clinical trials for multiple myeloma and myelodysplastic syndrome show efficacy and low toxicity in patients [178, 179]. The biological functions of RHAMM are complex. It is one of the first proteins to be identified for which its extracellular and intracellular functions differ markedly.

## 6. RHAMM Signaling

**Figure 4:**
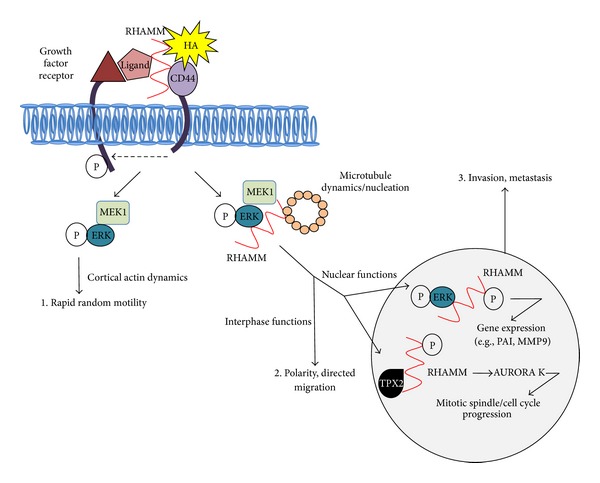
Model of RHAMM signaling. Model summarizes the known signaling functions of cell surface and intracellular RHAMM. Cell surface RHAMM interacts with CD44, HA, and growth factors to activate protein tyrosine kinase signaling cascades that activate the ERK1,2 MAP kinase cascade. This signaling can increase random motility in the absence of intracellular RHAMM. Intracellular RHAMM also binds to a number of protein partners that mediate its functions as a regulator of microtubule dynamics, centrosome structure/function, and gene expression. For example, during interphase, cytoplasmic RHAMM:protein partner interactions (MEK1/ERK1,2 shown) contribute to the dynamic properties of interphase microtubules and the number, placement, and structure of centrosomes, which affect cell polarity and direct cell migration. Nuclear RHAMM:MEK1:ERK1,2 complexes also control expression of genes involved in cell motility such as PAI-1 and MMP-9. During the cell cycle, RHAMM:TPX2 complexes contribute to mitotic spindle integrity and cell cycle progression through G2M while RHAMM:supervillin complexes promote cytokinesis.

The signaling functions of RHAMM are multifaceted and context dependent as might be expected by its complex subcellular compartmentalization. RHAMM is a cytoskeletal, centrosomal, mitotic spindle, and nuclear protein [49, 61, 166, 180], which is exported to the cell surface by unconventional mechanisms during wounding [181] by cytokines such as TGF*β*-1 [173]. Cell surface RHAMM associates with several integral protein tyrosine kinase and nonprotein tyrosine kinase receptors including PDGFR [182], TGF*β* Receptor-1 [170], CD44 [55, 64], CD44-EGFR complexes [183, 184], bFGFR [185], and RON [171]. RHAMM impacts upon the signaling competency of these receptors in response to their cognate ligands (Figure 4). Cell surface RHAMM:CD44 complexes, in association with one or more of the above growth factor receptors, promotes random cell motility in a protein tyrosine and ERK1,2 kinase dependent manner [186–188] (Figure 4). This random motility function does not require intracellular RHAMM proteins and immobilized recombinant cell surface RHAMM isoform (70 kDa) added to RHAMM−/−:CD44−/− fibroblasts is sufficient restore fibroblast motility speed to that of wild type or RHAMM-rescued fibroblasts [55]. Cell surface RHAMM also likely participates in functions required for wound repair such as cell division fidelity, mitotic spindle integrity, and cell cycle progression that were originally thought to be HA-independent functions of intracellular RHAMM proteins. For example, blocking cell surface RHAMM signaling reduces cell cycle progression of fibroblasts through G2M, a stage in the cell cycle where RHAMM and HAS2 mRNA are transiently elevated and for which HA production is necessary to facilitate cell rounding [189]. Exogenous HA also promotes the association of microtubule-associated protein homolog (TPX2) with nuclear RHAMM and phosphorylation of AURORA Kinase A (AURKA) to stimulate progression through the cell cycle [183]. The details of these signaling pathways as they are regulated by cell surface and intracellular RHAMM protein forms have been recently reviewed in detail [48, 61, 62]. The coordinated and separate signaling functions of intracellular and cell surface RHAMM in wound repair and in BCa remain to be resolved.

A simplified model of the proposed coordinated extracellular and intracellular RHAMM signaling functions is depicted in Figure 4. It is intriguing that both share the ability to regulate activation and subcellular localization of components of the MAP kinase (ERK1,2) cascade [48, 61, 62]. HA stimulation of cell surface RHAMM has consistently been shown to control the duration of ERK1,2 activity [55]. Intracellular RHAMM proteins form complexes with MEK1 and ERK1,2 and target these kinases to the cytoskeleton [165] and nucleus [55]. These signaling functions are required for random motility, mitotic spindle integrity, progression through the cell cycle, and gene expression (e.g., PAI-1 [170] and MMP9 [190]). RHAMM:ERK1,2 complexes are also likely to be important to centrosomal function since both RHAMM and ERK1,2 are required for microtubule nucleation [133, 191] and both are functionally linked to key centrosomal proteins such as TPX2 and AURKA [49, 183, 192]. In addition to microtubules, intracellular RHAMM partners with cortical actin proteins such as supervillin [193]. Supervillin, a membrane bound actin binding protein that participates in myosin II mediated contractility, interacts with calponin and regulates the activity of another RHAMM binding partner, ERK1,2 [194]. Supervillin coordinates processes that require dynamic cytoskeleton and membrane turnover including cell migration and cytokinesis [193, 195]. Indeed, RHAMM−/− cells often undergo aberrant cytokinesis causing the formation of multinucleated cells [165]. To date, the signaling functions of RHAMM can therefore be roughly divided into those that require (1) intracellular RHAMM and cell surface RHAMM (e.g., to control microtubule dynamics/nucleation and gene expression), (2) only cell surface RHAMM (e.g., to control random motility speed), and (3) only intracellular RHAMM (e.g., possibly cytokinesis).

While cell surface RHAMM controls the kinetics of ERK1,2 activation, intracellular RHAMM appears to target MEK1/ERK1,2 complexes to microtubules thus contributing to the dynamic turnover of interphase microtubules [196] and mitotic spindles [165]. Mitotic spindle formation is complex and includes key proteins such as AURKA and TPX2, which is a regulator kinase of AURKA. RHAMM-regulated ERK1,2 activity is required for bipolar spindle formation and loss of RHAMM can be compensated for by mutant active MEK1 in this function [165]. Intracellular RHAMM:TPX2 interactions and additional function interactions with BRCA1/BARD1 [49] also regulate the number, structure, and placement of centrosomes, in part through regulating AURKA activity. However, Hatano and colleagues have shown that nuclear RHAMM:TPX2 colocalization only occurs during metaphase. This group further showed that addition of HA stimulates both an association of RHAMM with TPX2 and an increase in the phosphorylation of the TPX2 regulator kinase, AURKA [183]. Since endogenous HA levels are high at G2M, it is likely that RHAMM:TPX2 interactions noted in other studies [49] are also controlled by HA. This centrosomal function of intracellular RHAMM is required for cell division fidelity in vascular response to injury, mitotic spindle integrity, progression through G2M, and basal-apical polarity of breast epithelial cells [57, 59, 197–199]. Nuclear RHAMM may play a further role in sequestering TPX2 [49] to these compartments to prevent premature changes in microtubules/mitotic spindle assembly and to facilitate repair of DNA aberrations caused, for example, by ionizing radiation [200]. In addition, RHAMM together with CD44 or TGF*β*R1, and possibly intracellular HA, may directly affect the transcription of genes controlling cell migration and proliferation [170, 190, 201] (e.g., PAI-1, MMP-9, Figure 4).

## 7. RHAMM Subcellular Compartmentalization

The mechanisms responsible for the complex subcellular targeting of RHAMM are still incompletely understood but are likely to be contributed to by isoform structure and posttranslational modification. RHAMM is subject to mRNA splicing and is phosphorylated by a variety of serine threonine kinases (Figure 3). Several RHAMM mRNA splice variants have been identified in breast and other cancers including a 48 bp deletion in exon 4 (RHAMM-48), a 346 bp deletion in exon 5, and a 147 bp deletion in exon 13 although the presence of these forms in wound repair has not been reported [202–205]. RHAMM is phosphorylated by protein kinase C, AURKA, and ERK1,2 [49, 197, 206]. Additionally, smaller than full-length N-terminal truncated RHAMM proteins have been reported during wound repair and in breast cancer cell lines [95]. Expression of these RHAMM isoforms and production of phosphoprotein specific antibodies have been utilized to identify isoform-specific subcellular targeting. These studies have shown that full-length RHAMM is largely associated with the cytoskeleton in interphase cells and in particular binds to microtubules [165, 207]. Targeting the nucleus is achieved by either alternative splicing of the full-length form in exon 5 [207], truncation of N-terminal sequence [208], or phosphorylation at T703 (human, but evolutionarily conserved [49]). The phosphorylation of RHAMM by protein kinase C*α* is required for rear-polarization of the microtubule organizing center (MTOC) of migrating neointimal smooth muscle cells [59, 197]. Cell surface labeling of cultured cells reveals a predominance of N-terminal truncated RHAMM proteins [209]. These small truncated proteins, which are generated by as yet unknown mechanisms, are less prominent on the cytoskeleton than the full-length protein, and FRAP studies show they are more mobile within the cell, accumulating in the nucleus and at the cell surface [61]. Since the multiple functions of RHAMM appear to be dictated by subcellular location it is likely that the various isoforms perform different functions and regulate distinct signaling pathways. Although isoform expression levels and interplay have been linked to tumor progression, their roles in response to injury and the differential manner in which they regulate specific functions are still poorly understood. The subcellular compartmentalization and signaling functions of RHAMM isoforms are critical for efficient repair of adult tissues and appear to provide some tumor cell types with growth, survival, and invasive advantages.

## 8. RHAMM and Tissue Remodelling

RHAMM mRNA and protein expression are coordinately and transiently upregulated following tissue injury. RHAMM protein expression is detected at the site of excisional skin wounds 24 hrs after injury, peaks at 3 days, and disappears by day 7 [55]. A similar rapid and transient increase in RHAMM expression is observed following scratch wound injury of fibroblasts and smooth muscle cells in cell culture [181]. Analyses of response to injury processes in RHAMM knockout mice or following functional blockade of RHAMM protein in wild type animals show that it regulates HA mediated ECM remodeling, polarized cell migration, cell division fidelity, and mesenchymal differentiation. These functions have been particularly well studied in vascular, lung, and excisional skin injury models [55, 57, 106, 119, 181, 197, 209, 210].

RHAMM was first shown to be required for smooth muscle cell migration into scratch wounds in the mid-1990s [181] and then later demonstrated to be required for endothelial cell signaling and migration during vessel morphogenesis in culture [123, 170, 185, 211]. More recently, the role of RHAMM was studied following vessel damage using RHAMM−/− mice [57, 119, 181, 197]. Cell culture studies comparing RHAMM−/− and wild type smooth muscle cells and blocking RHAMM function with antibodies show that RHAMM:HA interactions mediate smooth muscle cell adhesion and contraction of collagen gels.* In vivo*, loss of RHAMM increases vessel lumen size and reduces the size of adventitia and collagen deposition within the artery wall [57]. These results suggest that cell surface RHAMM:HA interactions promote lumen constriction and blocking this function of RHAMM may be clinically useful in preventing restenosis. The role of RHAMM in smooth muscle proliferation following balloon injury of rat carotid arteries has also been reported [197]. These studies show that the rapid proliferation of neointimal smooth muscle cells is RHAMM mediated. In this injury setting, RHAMM:dynein complexes localized to the mitotic spindle are required to promote mitotic fidelity by controlling centrosome placement. Furthermore, intracellular RHAMM phosphorylated by protein kinase C*α* is required for correct placement of centrosomes and directed migration of smooth muscle cells into wounds [59, 197]. In lung, RHAMM:HA fragment interactions are required for macrophage chemotaxis in surfactant-stimulated and bleomycin injured lungs [209]. RHAMM expression was first linked to skin wound fibroplasia and fibrosis* in vivo* using a transplantation model comparing incisional and excisional wounds [212]. The former heal without fibroplasia or scarring while repair of the latter is accompanied with extensive fibroplasia and scar formation. Only the excisional wounds exhibit increased CD44 and RHAMM expression. Later studies using RHAMM−/− mice, function blocking antibodies, and RHAMM mimetic peptide antagonists have established that HA:RHAMM interactions are critical for macrophage influx into the wound, as well as for fibroplasia and angiogenesis [55, 106]. Thus, blocking RHAMM function or deleting RHAMM expression results in a reduction of both M1 and M2 macrophages. Furthermore, loss of RHAMM function reduces the level of wound TGF*β*-1, causes reduced fibroblast migration into wounds, and inhibits their differentiation into myofibroblasts. There is also a reduction in collagen 1 accumulation and in the number of wound blood vessels.

Evidence from these studies and others [123] suggests that RHAMM binds to fragmented HA and that these interactions may be important in stimulating a RHAMM mediated “danger signal” to cells within injured tissues. Importantly, the binding of RHAMM to HA fragments is surprisingly size specific: a mixture of 4–20 saccharides promotes endothelial cell migration through RHAMM but HA-6 present in this mixture uniquely promotes wound closure, M1 and M2 macrophage influx into wounds, and TGF*β*-1 production through RHAMM and CD44 [56, 123]. In wound dermal cells, RHAMM:CD44 appears to cooperate to activate ERK1,2 and FAK. These results emphasize that HA-receptor interactions in healing wounds are complex and that multiple HA receptors can collaborate to control important aspects of wound repair. Similar RHAMM-regulated signaling appears to be at play in BCa progression.

## 9. RHAMM and Breast Cancer

To date, the role of RHAMM in BCa and other tumors have focused upon tumor cell parenchyma. However, it is likely that RHAMM expressed either by tumor or host cells directly or indirectly facilitates tumor progression. Blocking RHAMM in certain tumor cells inhibits tumor proliferation and migration/invasion while in others it primarily affects migration and invasion [48, 62]. RHAMM mRNA and protein expression are increased in most tumors and these high levels are positively associated with aggressive tumors. However, in a few tumor types (e.g., malignant peripheral nerve sheath tumors) knockdown of RHAMM levels actually enhances tumor aggression. Intriguingly, loss of RHAMM in these tumors is associated with increased AURKA activity and enhanced sensitivity to AURKA inhibitors [206, 213, 214]. RHAMM is also implicated in promoting the self-renewal and tumorigenic potential of tumor stem cells in cancers such as glioblastoma [215]. Despite the complexity of its functions in tumors, levels/distribution of RHAMM isoforms have diagnostic or prognostic value such as identifying which tumor types are sensitive to targeted therapy (e.g., AURKA inhibitors). Considering its involvement in many of the critical driver pathways important for malignant progression, the targeting of RHAMM may also have therapeutic value in some cancers including BCa (see below). For example, RHAMM silencing blocked the self-renewing capability of glioblastoma stem cells, and loss of RHAMM in malignant peripheral nerve sheath tumors or multiple myeloma enhances the sensitivity of tumor cells to AURKA inhibitors. RHAMM hyperexpression occurs in castration-resistant prostate cancer and is also associated with the likelihood of biochemical recurrence in prostate cancer patients with intermediate grade (Gleason grade 7) prostate tumors [206, 215–217].

Data bank indicates that hyperexpression of RHAMM mRNA expression is common in BCa and these elevated levels are often linked to poor clinical outcome [54]. Common genetic mutations at the low penetrance susceptibility RHAMM/HMMR locus enhance breast cancer risk in BRCA-1 mutation carriers [49]. Furthermore, in a large BCa patient cohort, RHAMM hyperexpression in breast tumor cell subsets predicts poor clinical outcome and is associated with elevated risk of peripheral metastases [218]. Other studies demonstrate that RHAMM expression is linked to increased BCa cell invasion and metastases [48, 62]. RHAMM transcription, which is regulated by mevalonate and HIPPO pathways, is required for ERK1,2-controlled BCa cell line migration and invasion, with relatively little impact on proliferation [96]. Similarly, RHAMM is an essential part of an autocrine motility mechanism in aggressive BCa lines for sustaining motility and invasion that requires HA production, ERK1,2 activation, and CD44 display [95]. Human BCa lines of all subtypes are heterogeneous in their ability to bind to fluorescent-HA probes. Subpopulations of tumor cells sorted according to their ability to bind to HA exhibit very different phenotypes. Cells that bind high levels of HA display both CD44 and RHAMM, proliferate slowly but are highly invasive in culture-based assay and* in vivo,* and are metastatic* in vivo*. In contrast, cell subpopulations, which bind low or no HA, express only CD44, proliferate rapidly but are poorly invasive in both culture based assays and* in vivo*, and are poorly metastatic [54]. These studies predict that major RHAMM functions in BCa are to support invasion and metastasis and that coordinated HA:cell surface RHAMM: intracellular RHAMM signaling contributes to BCa metastases in multiple but as yet incompletely understood ways [95, 165].

## 10. Conclusions

Wound repair and tumor progression are two complex but similar biological processes that share many molecular mechanisms for controlling cell migration, invasion, survival, and proliferation. HA and its receptors control essential functions in these two processes and this effect appears to be controlled in part by its binding to RHAMM. RHAMM is also upregulated during both processes where it appears to be similarly involved in the control of cell migration, invasion, proliferation, and differentiation. RHAMM is a multifunctional protein that signals through the ERK1,2 and TPX2 pathways at multiple steps. Its action on these pathways appears to be coordinately important to the initiation and progression of BCa and normal response to injury. The selective expression of RHAMM during times of tissue remodeling makes it a promising marker and target for diagnosis and therapy of disease involving aberrant wound repair and cancer.

## Abbreviations

AURKA: AURORA Kinase A
BCa: Breast cancer
CD44: Cluster designation 44
ECM: Extracellular matrix
ERK1,2: Extracellular regulated kinase 1,2
HA: Hyaluronan
HAS: Hyaluronan synthase
Hyal: Hyaluronidase
HMMR: Human HA mediated motility receptor
MMP-9: Metalloproteinase-9
PAI-1: Plasminogen activation inhibitor 1
RHAMM: Receptor for HA mediated motility
TLR2,4: Toll-like Receptor 2,4
TPX2: Microtubule-associated protein homolog.
